# Photophysical deactivation behaviour of Rhodamine B using different graphite materials†

**DOI:** 10.1039/c9ra03325d

**Published:** 2019-07-18

**Authors:** Varnika Prakash, Rekha Bhar, Shweta Sharma, S. K. Mehta

**Affiliations:** Institute of Forensic Science and Criminology, Panjab University Chandigarh 160014 India; Department of Chemistry, Centre of Advanced Studies, Panjab University Chandigarh 160014 India skmehta@pu.ac.in

## Abstract

In the present work, an attempt has been made to elucidate the structural features of synthesized graphite materials, *i.e.*, expanded graphite (EG) and an expanded graphite/silver nanoparticles (EG/AgNPs) nanocomposite. In order to obtain knowledge about the functional groups present, the interlayer spacing between the carbon layers, topographical features, and the characterization of the materials were carried out using Fourier-transformer infrared spectroscopy, X-ray diffraction, Raman spectroscopy, field emission scanning electron microscopy-energy dispersive X-ray spectroscopy and atomic force microscope. Furthermore, the quenching efficiency of the synthesized graphite materials was also compared using Rhodamine B (Rhd B) as a fluorescent probe. The non-linear behaviour of the Stern–Volmer plots suggested that the complex quenching mechanism (a combination of static and dynamic quenching) was responsible for the decrease in photoluminescence intensity. At a lower concentration of the quencher, the static quenching mechanism was dominant whereas at a higher concentration dynamic processes seemed to be more likely. The binding strength of the complexation between the fluorophore and the quencher at lower concentrations was studied in detail for both of the synthesized materials. The analysis showed that the EG/AgNPs exhibited better quenching efficiency and possessed a strong binding strength in comparison to EG. The thermodynamic parameters of this association suggested that the interaction process was spontaneous and exothermic in nature. Thus, this work offers helpful insights into the fluorescence quenching mechanisms of the Rhd B/EG and its composite system.

## Abstract 2

Graphical representation of varying quenching mechanism of RhD B dye using different graphite materials *i.e.* EG and EG/AgNPs.

## Graphical abstract

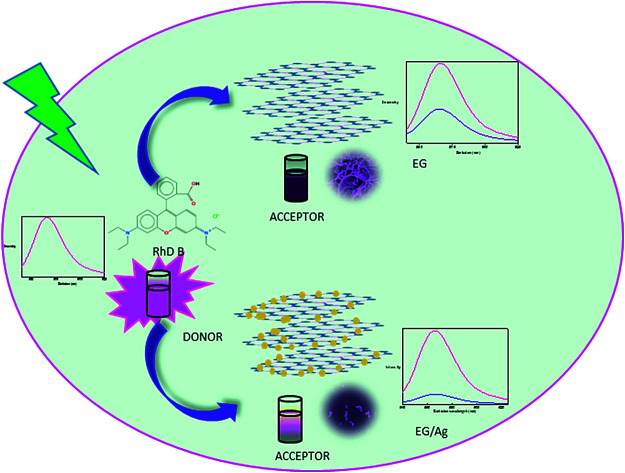

## 1. Introduction

The potential of graphene-based materials namely, graphene oxide (GO),^1,2^ reduced graphene oxide (rGO),^3^ and carbon nanotubes^4^ as efficient fluorescence (FL) quenchers have been explored extensively. Another related member of the graphene family that falls into the graphene intercalated compounds is expanded graphite (EG), also known as “intumescent flake graphite”. The EG and its integrated metal oxides or polymer composites are also gaining much attention for their use in thermal energy storage,^5^ sensors,^6^ medical dressings,^7^ antistatic, and anti electromagnetic^8^ materials. The EG is generally obtained by intercalating molecules and atoms such as aluminium (Al), hydrogen peroxide, lithium, phosphoric acid, potassium, sodium, sulfuric acid (H_2_SO_4_), followed by heat treatment.^9^ The product obtained is around 300 times more voluminous, with an approximately 10-fold increase in the surface area as compared to the graphite precursor.^10^ Zhao *et al.*^11^ synthesized EG embedded with Al nanoparticles for use in high-performance lithium-ion batteries. Jovic *et al.*^12^ functionalized iron oxide nanocrystals into exfoliated EG and studied their structural details using spectroscopic techniques. The findings revealed that defects present on the exfoliated graphene sheets acted as anchoring sites for adsorption of the nanocrystals but the adsorption was non-preferential in terms of the adsorption sites. Silver nanoparticles (Ag NPs) have been chosen previously, for synthesizing EG composites because of their active use in antimicrobial, therapeutic and diagnostic platforms.^13^

In various studies, FL is the most commonly used method to investigate the structural features of graphene-based materials. Evaluation of emitted radiation can reveal not only information about the structural features^14^ but also the molecular interactions with the fluorophores^15^ and the thermodynamics of the system.^16^ Generally, these studies are used for sensing and detection of ionic species,^17,18^ biomolecules,^19^ and hazardous chemicals.^20^ A quantitative analysis of the quenching efficiencies of various graphene materials has been carried out, which explained how chemically exfoliated rGO was better than graphite and GO.^21^ Liu *et al.*^22^ investigated the electron transfer efficiency of GO and graphene using different dyes, namely, eosin, Rhodamine B (Rhd B) and Methylene blue. It was observed that the rate of electron transfer was much higher and efficient for graphene because of its stronger electrostatic interactions with dyes. Lu *et al.*^23^ also compared GO and rGO for a better adsorption capability and sensing of fluorescent labeled DNA.

The electron transfer or Förster resonance energy transfer (FRET) processes are known to be responsible for the quenching capability of graphene materials.^24^ The graphene material acts as an energy acceptor that quenches the FL of an energy donor. Lin *et al.*^25^ stated that strong London dispersion forces were responsible for polarization and closeness in the graphene layers and organic dye molecules. The quenching mechanism involved electron transfer from the dye (donor) to the graphene (acceptor) involving intimate π–π interactions. The thermodynamics of the quenching system can provide significant information, as it categorizes the quenching mechanism to be either static or dynamic and also describes the nature of the reaction. Static quenching, which results because of a ground state complex formation between the fluorophore and the quencher, gives information about the binding strength and tertiary or quaternary structural changes in the macromolecule.^26^ The static interactions are stronger when compared to the random weak collisional dynamic ones. The experiments, and their results, described in this paper are an attempt to unravel the structural details, and the binding strength of the synthesized material with the fluorophore. The thermodynamics involved during the quenching of Rhd B as a fluorophore by the synthesized EG and its composite were also studied.

## 2. Experimental

### 2.1 Chemicals and instrumentation

All the chemicals used in this study were of analytical grade. Graphite flakes (>3 μm), Rhd B, ascorbic acid (99%), poly(vinylpyrrolidone) (PVP) (MM 40 000), and *N*-propanol (anhydrous, 99.7%), were purchased from Sigma-Aldrich. Silver nitrate (AgNO_3_, 99.8%), was obtained from SD Fine-Chem, and H_2_SO_4_ (98%), nitric acid (69–70%) were obtained from Finar Ltd., and Fisher Scientific, respectively. Double distilled water was used for solution preparation and washing purposes.

The Fourier-transform infrared (FTIR) spectra of the synthesized materials were recorded using potassium bromide pellets on a RZX FTIR spectrophotometer from (PerkinElmer) in the mid-infra-red region (4000–400 cm^−1^). A D8 Advance X-ray diffractometer (Bruker) equipped with a Cu-Kα radiation source (*λ* = 1.54 Å) under an accelerating voltage of 40 kV and 25 mA for a 2*θ* range of 5–50° was used for recording X-ray diffraction patterns (XRD) patterns. The field emission-scanning electron microscopy (FESEM) images were recorded using a SU8010 emission scanning electron microscope (Hitachi). The energy-dispersive X-ray spectral analysis (EDX) used for the elemental mapping of EG and EG/AgNPs was performed in a confined region of 100 nm using a Bruker EDX analyzer. The quantitative elemental the analysis of elements such as carbon (C), nitrogen (N) and oxygen (O) in both the prepared materials were performed.

Raman spectroscopic analysis was carried out using an ISA LabRam-300 Raman spectrometer (Horiba Scientific). The excitation of the helium-neon laser was 538.2 nm with power of 10 mW and about a 1–2 μm laser spot size.

The atomic force microscopy (AFM) studies were conducted using a di-Innova atomic force microscope (Veeco Instruments) in tapping mode equipped with a silicon substrate and cantilever, a resonance frequency of 300 kHz and a spring constant of 60 N m^−1^. The FL spectra were recorded using an F-7000 FL spectrophotometer (Hitachi).

### 2.2 Preparation of EG and EG/AgNPs

#### 2.2.1 Preparation of EG

Graphite flakes (2 g) were kept overnight in a mixture of H_2_SO_4_ and nitric acid in a 3 : 1 ratio. The treated flakes were then repeatedly washed with distilled water and then dried at 60 °C for 12 h. The conversion of these graphite flakes into EG was done by heating the flakes in a conventional microwave for less than a minute. The flakes were observed to expand and turn into black, fluffy, worm-like structures (Fig. S1, ESI†).

#### 2.2.2 Preparation of EG/AgNPs

As the synthesized EG was found to be insoluble in an aqueous system, and the mixture of ethanol and nitro compounds were known to be explosive in nature, propanol was chosen as the solvent for the preparation of the EG/AgNPs. The EG dispersion (1 mg ml^−1^, 20 ml) was prepared using ultrasonic mixing for 1.5 h. Ascorbic acid (40 mg) and PVP (0.27 g) were added one after the other into the dispersion with stirring at room temperature. Subsequently, the AgNO_3_ (0.067 g) was added and the dispersion was mixed ultrasonically for 10 min followed by stirring for 5 h. The dispersion turned from black to grey in color, and was then washed with distilled water and ethanol and dried at 60 °C.

#### 2.2.3 Fluorescence measurements

The Rhd B (0.5 μg ml^−1^ in propanol) was used as a fluorophore and its FL spectrum was recorded at an excitation wavelength (exc *λ*) of 510 nm and the emission wavelength was obtained at (em *λ* 565 nm). A stock solution of the graphene material was prepared in propanol and 100 μl aliquot of this stock solution was added to the Rhd B solution. The solution was stirred well before recording each spectrum. Additions were made until the equilibrium point was reached.

#### 2.2.4 Characterization

**Fig. 1:**
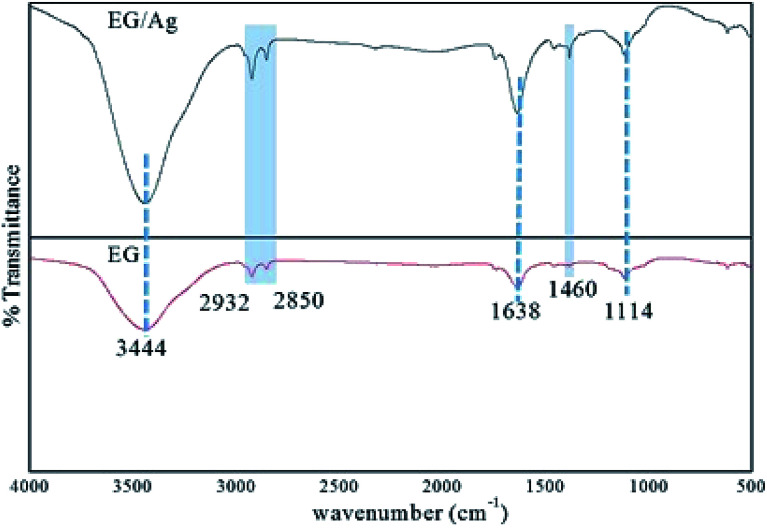
FTIR spectra of EG and EG/Ag showing the presence of polar functionalities on the surface of the synthesized materials.

The FTIR spectrum for EG showed the presence of polar groups on the graphite surface. The peak around 3444 cm^−1^ corresponded to the O–H stretching mode, and the two small peaks around 2932 cm^−1^ and 2850 cm^−1^ were designated as the C–H stretching in the alkanes and aldehydes (=C–H), respectively. Furthermore, the peaks seen at 1638 cm^−1^ and 1114 cm^−1^ corresponded to the C=C stretching introduced into the graphitic carbon network and C–O vibration mode of enols, respectively. In addition to these peaks, a small kink present at 1745 cm^−1^ represented C=O stretching in the carbonyl and carboxyl groups. The vibrations engaging the C and O indicated the presence of oxygen functionalities even after the microwave treatment for exfoliation of the graphite layers. Furthermore, a weak stretching frequency observed in the fingerprint region (around 1460 cm^−1^) could be assigned to the aromatic C=C bonding. The spectrum of EG/AgNPs showed peaks at a similar wavenumber to that of EG, suggesting that Ag NPs did not affect the structural characteristics of EG. However, change in the relative intensity of certain bands in the spectrum of the EG/AgNPs indicated the association of AgNPs onto the EG surface. A peak at 3444 cm^−1^ was seen to become sharper, and was attributed to the hydrogen bonding between the AgNPs and EG (Fig. 1). A major change can also be seen in the aromatic C=C stretch. This peak became more prominent in the presence of AgNPs, indicating that there was a π–π interaction between EG and the AgNPs.^27^ Integration of the AgNPs onto the surface of EG prevented the effective agglomeration of NPs, thus providing enhanced stability to the system.

**Fig. 2:**
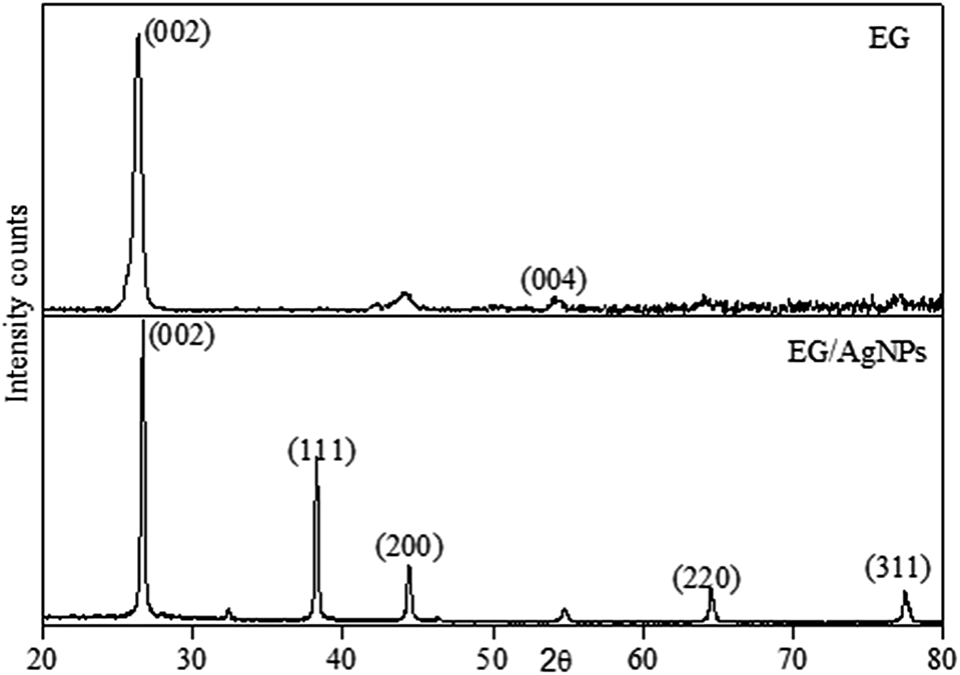
XRD pattern of EG and EG/AgNPs.

The XRD pattern obtained for EG showed a peak at a 2*θ* of 26.3 which was a characteristic graphitic peak corresponding to the (002) plane. This peak was also seen in EG/AgNPs with a very slight shift at a 2*θ* of 26.7. The shift can be ascribed to the change in diffraction angle because of the presence of the AgNPs. The smaller peak seen for EG at 54.1° was ascribed to (004) graphitic plane. However, the additional sharp peaks at 38.1°, 44.5°, 64.5°, and 77.6° were observed for the EG/AgNPs, which were assigned to silver crystalline planes (111), (200), (220), (311) of face centred cubic (fcc) unit cell, respectively (Fig. 2).

The corresponding interlayer distance in EG and the EG/AgNPs composite was estimated to be 3.39 Å and 3.29 Å, respectively, using Bragg's equation. The positive interaction between the AgNPs and EG were responsible for the closer proximity of the two EG layers, thus, a smaller interlayer distance was observed for the EG/AgNPs. The calculated average crystallite size of the AgNPs using the Debye–Scherrer's eqn (1) was 31.7 nm.1
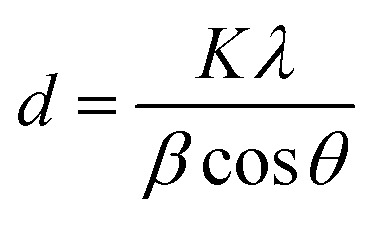
where, *d* is the crystallite size, *K* is a dimensionless shape factor, *λ* is the X-ray wavelength, *β* is full width at half maxima and *θ* is Bragg's angle.

**Fig. 3:**
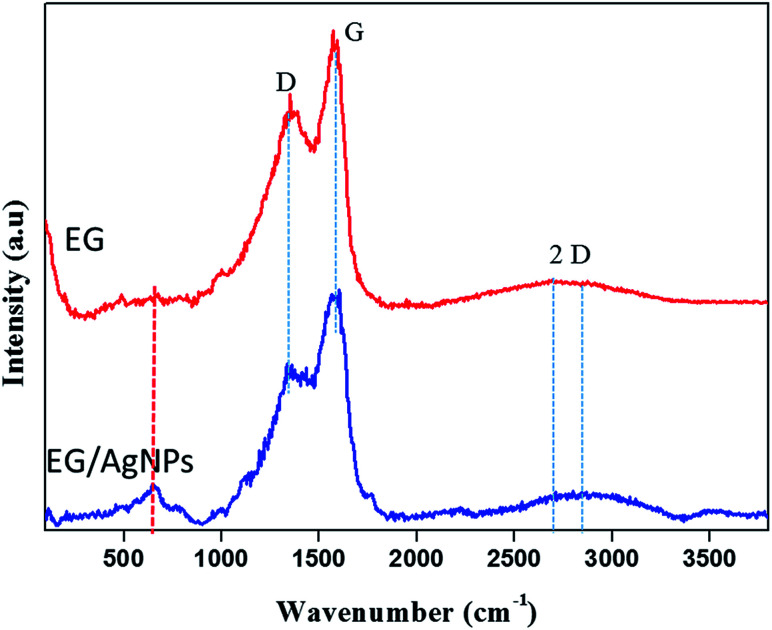
Raman spectra of EG and EG/AgNPs showing the D, G, and 2D peaks. The peak marked in red for the EG/AgNPs samples indicates the presence of AgNPs.

The results of the Raman analysis show that the graphitic carbon nature of the EG was denoted by the G peak seen at about 1570 cm^−1^ and a peak at about 1350 cm^−1^ suggests that there are covalently attached oxygen functionalities such as epoxide on the EG surface^28^ (Fig. 3). Another broadened two-dimensional (2D) peak at about 2700 cm^−1^ was seen in EG which tended to shift in the case of EG NPs. The broadening of the 2D peak was an indication of a multiple layer graphene structure. In both cases, the broadened 2D peak was suggestive of multi-layer graphene, with a shift and an additional peak at about 653 cm^−1^ which was because of the incorporation of AgNPs on the EG surface.^29^

**Fig. 4:**
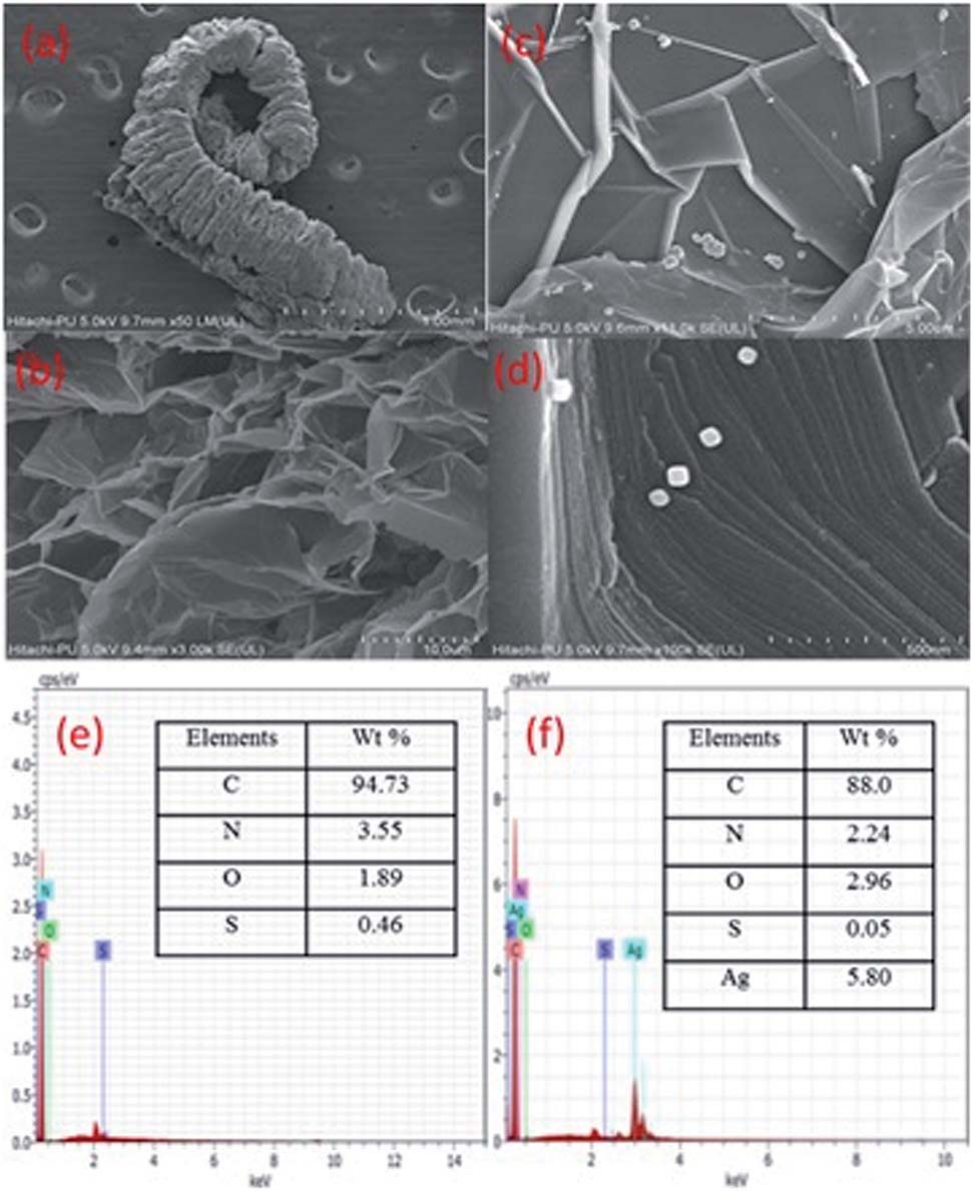
FESEM images of a single expanded graphite flake showing a worm like structure (a), a close view of the exfoliated sheets in EG after microwave treatment (b), a larger view of adsorbed Ag agglomerates and particles on the surface of EG (c), well defined cubical AgNPs seen on the EG surface (d), and EDX patterns of EG (e) and EG/AgNPs (f).

The FESEM images (Fig. 4) show exfoliated sheets for EG which were morphologically different from that of pristine graphite flakes (Fig. S2, ESI†). The microscopic images of graphite show closely stacked multi-layered carbon sheets, whereas, EG shows exfoliated carbon sheets. The tendency of intercalated acid molecules to escape increased because of the pressure generated during the heating (microwave) process. This resulted in an expansion of the adjoining carbon layers. The intercalated acid molecules after the microwave treatment were evolved in the form of gases such as nitrogen dioxide and sulfur dioxide. However, the EG/AgNP images show that the AgNPs were adsorbed on the EG surface. The shape of the AgNPs was cuboidal and the size varied from 15 nm to 30 nm. The AgNPs were prone to agglomeration because of the active surface plasmon resonance,^30^ however, in the present case minimum aggregation was observed. The exfoliated carbon layers acted as a substrate for the efficient adsorption of AgNPs resulting in their fine dispersion. In addition, PVP also assisted in stabilizing and governing the shape and size of the particles.

The EG/AgNPs images show that the AgNPs were adsorbed on the EG surface. The shape of the AgNPs was cuboidal and the size varied from 15 nm to 30 nm. The exfoliated carbon layers acted as a substrate for an efficient adsorption of AgNPs, resulting in their fine dispersion. In addition, PVP also assisted in stabilizing and governing the shape and size of the particles.

Furthermore, the EDX analysis, confirmed that the major elemental composition was: C, O, S, and N for the EG together with additional Ag for the EG/AgNPs. The corresponding elemental percentage of C, O, S, N in EG was 94.3%, 1.89%, 0.46%, 3.55%, respectively, whereas in the EG/AgNPs it was 88%, 2.96%, 0.55% and 2.24%, respectively, with 5.80% of Ag.

**Fig. 5:**
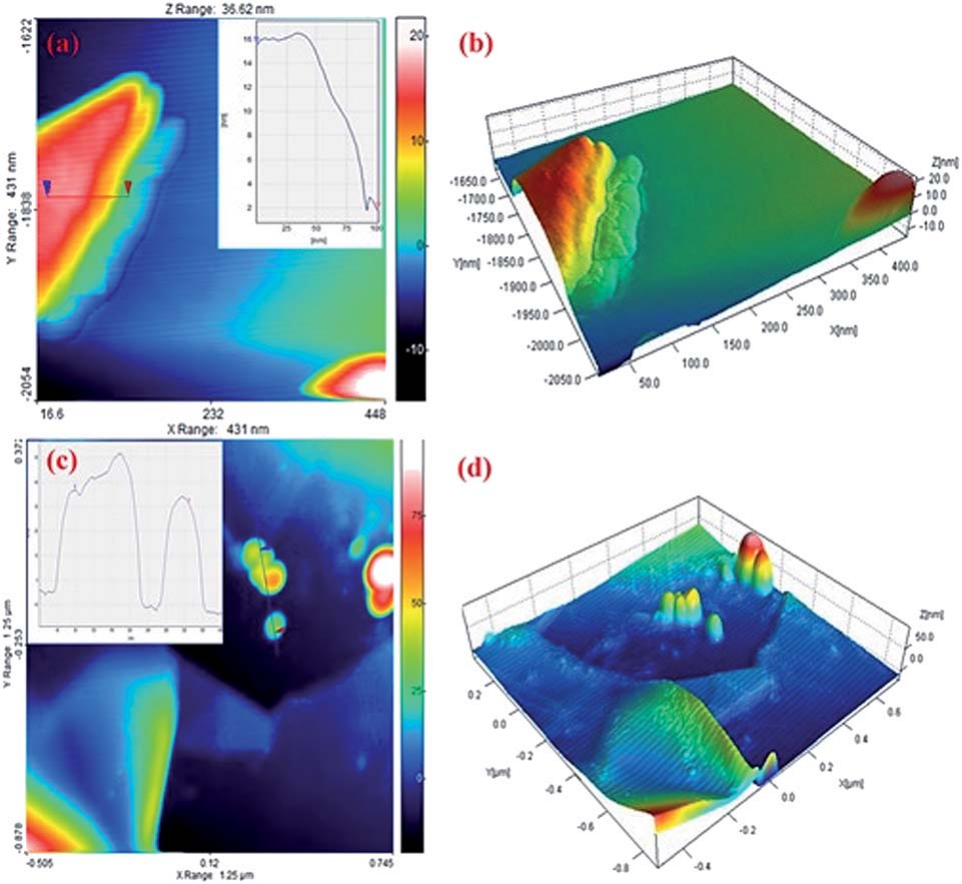
(a and b) AFM image and height profile of EG (c and d) EG/AgNPs on a silicon wafer.

AFM was used to measure the graphite sheet size and thickness. The EG and EG/AgNPs were mixed ultrasonically and dispersed in propanol. A portion (20 μl) of this solution was drop casted over a previously cleaned silicon wafer and dried at room temperature. The thickness of the carbon sheet at the blue mark was about 16 nm which drastically decreased to 3 nm near the edges (red mark, Fig. 5). This can be corroborated by the fact that the intercalated acid molecules at the edges were loosely bound when compared to the molecules in the centre, and thus could easily escape. As a result of this, the layer at the edges expanded to a greater extent. The AFM results obtained were in good agreement with the results of the FESEM and XRD analysis. Also, the height profile analysis showed that nanosheets of 12 nm mean thickness were present in the EG sample.

## 3. Fluorescence (FL) studies

**Fig. 6:**
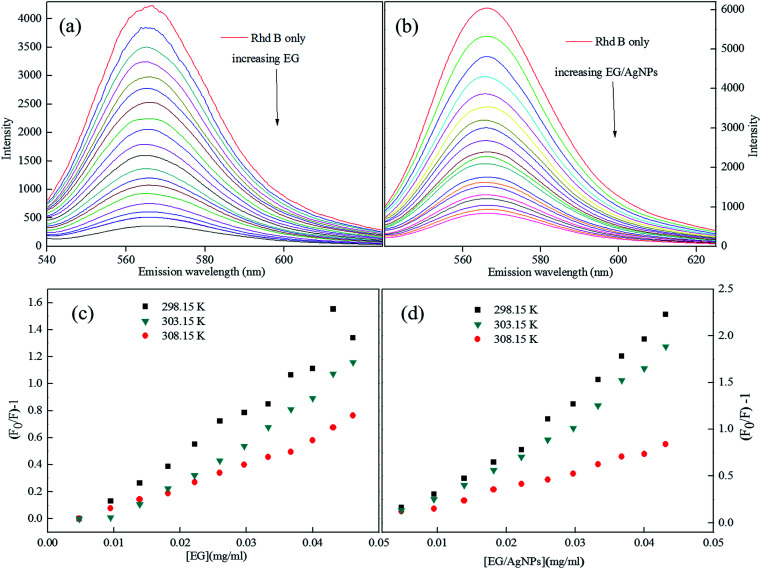
(a) FL plots of Rhd B in presence of EG, and (b) EG/AgNPs, (c and d) corresponding Stern–Volmer plots (lower concentration range) at different temperatures.

The Rhd B shows a prominent FL peak centred at 565 nm. However, the subsequent additions of EG and EG/AgNPs (0.2 mg ml^−1^) lead to a decrease in FL intensity with no shift in *λ*_max_ (Fig. 6a and b).

**Fig. 7:**
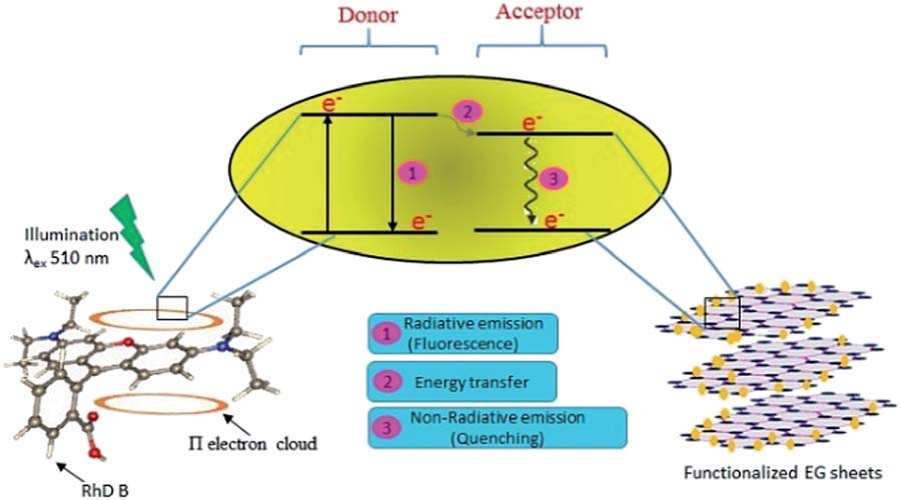
Schematic representation of the likely mechanism of the photodeactivation process of Rhd B on the introduction of the functionalized EG sheets.

An absence of shift in *λ*_max_ confirmed that the quenchers do not cause any structural change in the Rhd B fluorophore. Positive interactions of EG and EG/AgNPs with the conjugated π electron cloud of the dye lead to photophysical intermolecular deactivation of the process. Functionalities such as carbonyl, ethanolic, ethylenic and enolic groups present on the surface of EG and EG/AgNPs can easily interrupt the conjugation of the π electron cloud of the dye by electrostatic interactions. The functional groups present on the surface of EG and EG/AgNPs act as energy acceptors, thus, providing an alternative non-radiative electron transfer pathway.^18^ Comparing the two, the EG/AgNPs seem to be a more potent quencher than EG alone (Fig. 7).

In order to obtain further information, Stern–Volmer (SV) plots were drawn (Fig. S2, ESI†). A plot between (*F*_0_/*F*) − 1 *versus* concentration (eqn (2)) showed an upward curvature which indicated a dual quenching mechanism, *i.e.*, static as well as dynamic.2
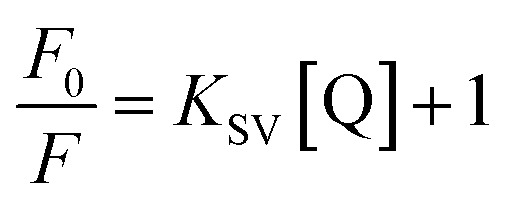
where, *F*_0_, *F*, *K*_SV_, and [Q] are the intensity of the fluorophore in the absence and presence of quencher, the SV constant, and the quencher concentration, respectively.

Fig. S2 (ESI)† can be segregated into two linear segments before and after the inflection point (yellow mark), one being at a lower concentration range (0.0048–0.046 mg ml^−1^) and another at a higher concentration range (0.048–0.066 mg ml^−1^). The decrease in FL intensity was gradual at a lower concentration range in comparison to a prominently sharp change at a higher concentration range. At a lower concentration, the probability of complex formation between Rhd B and the quencher molecule (EG and EG/AgNPs) was very high. However, at a higher concentration, this probability decreased because of extensive inter-particle collisions. Thus, the lower concentration range can be designated as a static quenching mechanism, whereas at a higher concentration, it was a dual quenching mechanism, *i.e.*, a static, as well as dynamic process, is followed.^31,32^ To determine the binding strength, a linear fit was applied to the static region of the SV plots (Fig. 6c and d).

The value of *K*_SV_ which was analogous to the binding strength is listed in Table 1. The interactive forces between Rhd B and the quencher are stronger for the EG/AgNPs when compared to EG as was suggested by the higher *K*_SV_ values. The presence of Ag NPs may provide more active sites to interact with Rhd B, thus making the EG/AgNPs a stronger binder. Furthermore, with an increase in temperature, *K*_SV_ values decreased in both cases. Lowering of the binding strength with an increase in temperature can be allocated to the fact that the higher temperature may weaken the interaction forces by providing heat energy to the system.

**Table 1:** The *K*_SV_ values for EG and EG/AgNPs at different temperatures

Temp (K)	*K*_SV_ (L g^−1^)	
	EG	EG/AgNPs
298.15	28.60	46.18
303.15	21.18	18.71
308.15	14.31	10.21

### 3.1 Thermodynamics of the system

To investigate the thermodynamics behind the process, the quenching process was studied at three different temperatures and the data was interpreted using the Van't Hoff equation:3
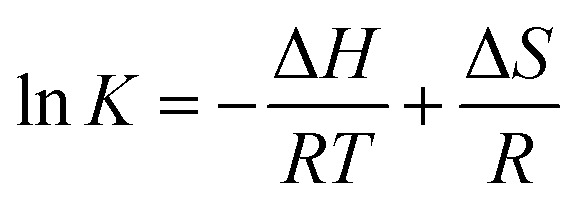
where *K* is an equilibrium constant, Δ*H* is the enthalpy change, Δ*S* is the entropy change, *R* is the universal gas constant, and *T* is the temperature in Kelvin. The value of Δ*H* and Δ*S* can be determined from the slope and intercept of the plot between ln *K versus* 1/*T*, respectively. Knowing the value of Δ*H* and the Δ*S* Gibbs free energy (Δ*G*) of the system can be evaluated as follows:

4 Δ*G* = Δ*H* − *T*Δ*S*

The parameters obtained from the Van't Hoff equation are shown in Table 2.

**Table 2:** Thermodynamic parameters of the quenching process for both the systems

*T* (K)	Δ*G* (kJ g^−1^)		Δ*H* (kJ g^−1^)		*T*Δ*S* (J)	
	EG	EG/AgNPs	EG	EG/AgNPs	EG	EG/AgNPs
298.15	−8.355	−9.365	−52.848	−115.414	−44.491	−106.605
303.15	−7.608	−7.587			−45.232	−107.827
308.15	−6.862	−5.808			−45.982	−109.605

**Fig. 8:**
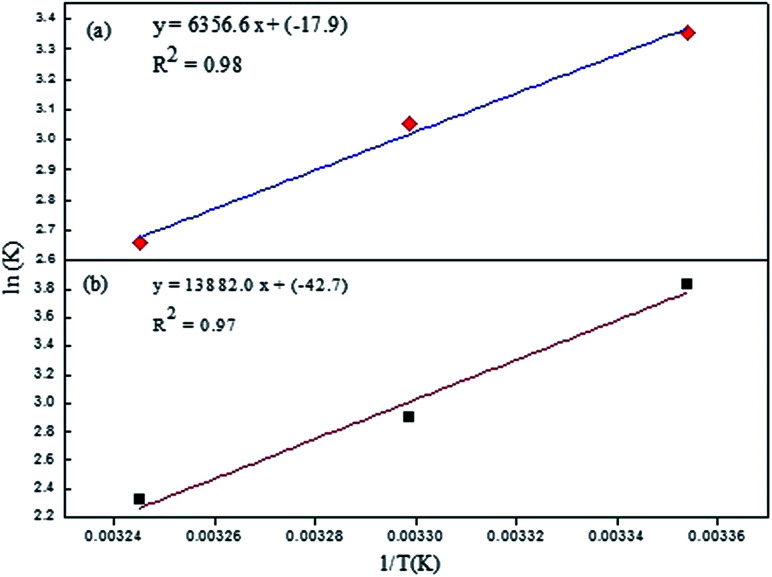
Van't Hoff plots of (a) EG and (b) EG/AgNPs.

A negative value of Δ*G* indicated that the interaction process between Rhd B and the quenchers was spontaneous. At a higher temperature, the process became less probable as shown by the lower value of Δ*G*. Also, a negative value of enthalpy changes suggested that heat energy was evolved during the interaction or that the process was exothermic in nature. The results were in agreement with the non-radiative emission of the energy during the quenching. The positive interactions between the two species lead to the closer proximity of the two, and as a result, the randomness of the system decreased. This fact was also supported by the negative value of Δ*S*. Comparing the two systems, the interaction of EG/AgNPs with Rhd B was more spontaneous which was also in good agreement with the SV constant and other findings. All the parameters reinforced the likely interaction between Rhd B and EG and its conjugate with AgNPs (Fig. 8).

## 4. Conclusions

Structural and morphological features of the synthesized materials, *i.e.*, EG and EG/AgNPs have been determined and described. Microscopic studies revealed that the carbon sheets tended to exfoliate from the corners providing a bed for the cuboidal AgNPs to adsorb to. Spectroscopic studies showed the introduction of polar entities on the carbon sheets after the microwave treatment of acid-treated graphite. The crystallinity of AgNPs could be determined from the sharp and clear peaks obtained from the XRD pattern. Furthermore, the interactions of the materials prepared using the fluorophore, Rhd B were studied using FL spectroscopy. The results demonstrated the different complex formation for both the materials. The EG/AgNPs bind with the Rhd B molecules with a higher binding strength than EG because of the increased adsorption sites, better energy transfer between the donor (Rhd B) and the acceptor (EG). The thermodynamics of the system suggested that the reaction was spontaneous and exothermic in nature.

## Conflicts of interest

There are no conflicts to declare.

## Supplementary Material

RA-009-C9RA03325D-s001
