# Supplementary material for: Photophysical deactivation behaviour of Rhodamine B using different graphite materials
Source: RSC Adv. 2019 Jul 18;9(39):22320–6. doi: 10.1039/c9ra03325d (PMC9066896; doi:10.1039/c9ra03325d)
Supplement: RA-009-C9RA03325D-s001 [file RA-009-C9RA03325D-s001.pdf]

Electronic Supplementary Material (ESI) for RSC Advances.
This journal is © The Royal Society of Chemistry 2019

Supplementary Information

Photophysical deactivation behaviour of Rhodamine B by different graphite materials

Varnika Prakash[a], Rekha Bhar[b], Shweta Sharma[a] and S.K.Mehta[*b]

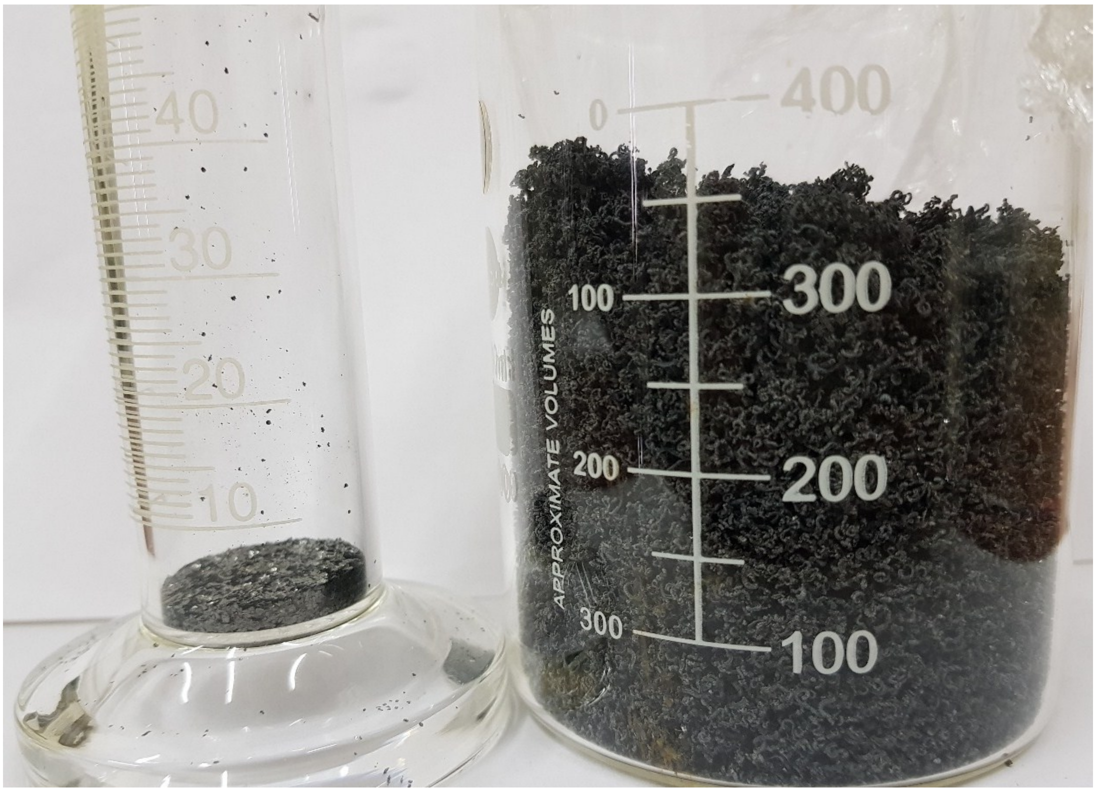

Fig. S1 Photographic image of graphite (left) and EG(right)

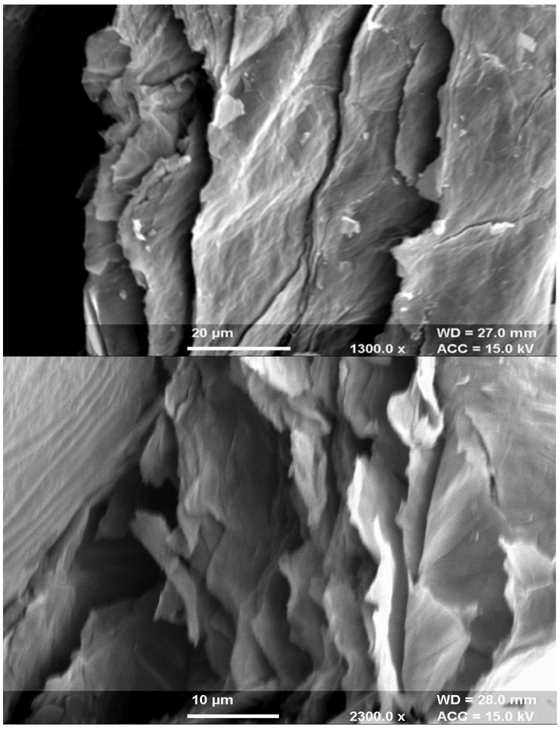

Fig. S2 FESEM images of graphite flakes

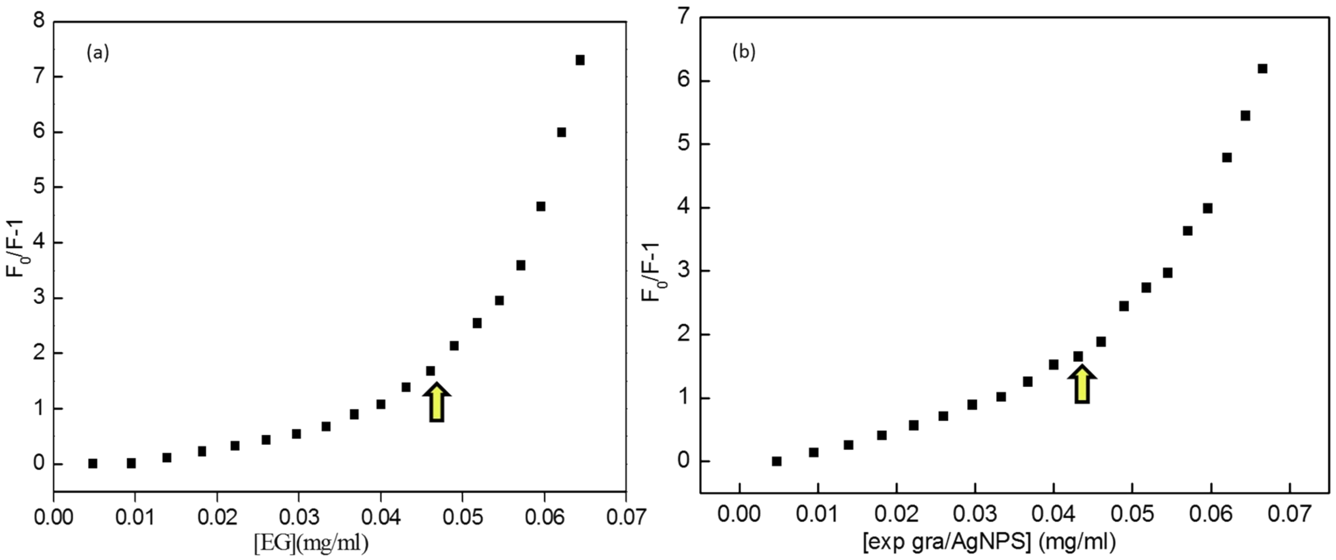

Fig. S3 (a) Stern-Volmer plot of Rhd B-EG system (b) Rhd B-EG/AgNPs system at 303.15 K
